# The undeveloped properties of GABA neurons in the ventral tegmental area promote energy intake for growth in juvenile rats

**DOI:** 10.1038/s41598-019-48336-5

**Published:** 2019-08-14

**Authors:** Yuko Maejima, Shoko Yokota, Shoichiro Horita, Kenju Shimomura

**Affiliations:** Department of Bioregulation and Pharmacological Medicine, Fukushima Medical University School of Medicine, Fukushima, 960-1295 Japan

**Keywords:** Hypothalamus, Hypothalamus

## Abstract

Juvenile animals show higher energy intake (EI) per body weight (BW) to meet the energy requirements for growth. However, the underlying mechanisms that induce high EI/BW in juvenile animals remain unknown. The EI from a control diet (CD) and high fat diet (HFD), as well as BW changes were compared between juvenile (3 weeks old) and adult (8 weeks old) rats. BW gain and EI were increased in the HFD-fed adult rats compared to the CD-fed adult rats. However, in the juvenile rats, there were no differences in BW gain and EI between the CD-fed and HFD-fed groups. The locomotor activity was significantly increased in HFD group compared with the CD group in juvenile, but not in adult rats. Gamma-aminobutyric acid (GABA) neurons in the VTA were found to remain undeveloped with less GABAergic input into dopamine neurons in the juvenile rats. The deletion of the VTA GABA neurons in the adult rats significantly increased CD consumption, but showed almost no change in HFD consumption. These data suggest that undeveloped properties of VTA GABA neurons in juvenile rats can promote higher EI regardless of high or less palatable feeding, and contribute to growth promotion.

## Introduction

The neonatal and juvenile periods are critical stages of development during the growth stage^1–3^. In these periods, animals, including humans, need to maximize energy intake (EI) in order to meet the energy requirements to achieve growth and survival. However, little is known about the mechanisms that enable increases in EI in juvenile animals.

In adult animals, total EI is regulated by two mechanisms. One is homeostatic food intake and the other is hedonic/reward food intake. These two mechanisms are regulated in two different areas of the brain, the hypothalamus and limbic system^4,5^. The hypothalamic arcuate nucleus (ARC) is a critical component that regulates homeostatic EI and body weight (BW)^6,7^. The projections from ARC to other hypothalamic nuclei, such as the paraventricular nucleus (PVN), dorsomedial hypothalamic nucleus (DMH), lateral hypothalamic area (LHA) and ventromedial hypothalamic nucleus (VMH), are important in regulating EI and BW^4,8^.

On the other hand, the limbic system plays the role of reward-related feeding^9^, such as consumption of high palatable food. The ventral tegmental area (VTA) dopamine neurons are known critical mediators in food reward^10^, and the projection from dopamine neurons to the nucleus accumbens (NAc) promote the initiation and maintenance of reward seeking and consumption^9–11^.

VTA is a heterogeneous brain structure containing a neural population such as dopaminergic (~65%), GABAergic (~30%) and glutamatergic (~5%) neurons^12–15^. The gamma-aminobutyric acid (GABA) neurons distributed in the VTA directly suppress the activity and excitability of neighboring dopamine neurons, as well as release of dopamine in the NAc^12^.

The properties of the hypothalamus and limbic pathway at the juvenile stage are reported to be different compared to those of adults. In the hypothalamus, it is reported that the projection from ARC to PVN or DMH are immature at birth, and ARC circuits are structurally and functionally immature until the third week of postnatal life in mice^16^. Thus, it is considered that the hypothalamic feeding circuit is established at the weaning stage in mice. As for the limbic pathway, a previous study showed that anorexigenic adipocytokine, leptin, which is reported to reduce the firing rate of VTA dopamine neurons in adult rats, failed to increase leptin signaling in dopamine neurons in rat VTA at postnatal day 16^17^. Additionally, dopamine neurons in the VTA fire faster in five- to seven-week-old adolescent rats than those of 12- to 15-week-old adult rats^18^. These above mentioned reports suggest that VTA neurons are functionally immature in juvenile rats, but develop with growth and age. However, compared with the hypothalamus, the detailed mechanism of development in the reward-related limbic system remains unknown.

The aim of the current study was to clarify the characteristics of feeding regulation in the limbic system of juvenile rats. We herein show the immaturity of GABA neurons and the decreased inhibitory input into the dopamine neurons in juvenile rat VTA to confirm the increase of EI, regardless of high or low palatable food. The present study not only investigate the mechanism for promoting growth in juvenile animals but may also provide a better understanding of the mechanism for the development of hyperphagia in obese children.

## Results

### The characteristic of feeding regulation in juvenile rats

**Figure 1:**
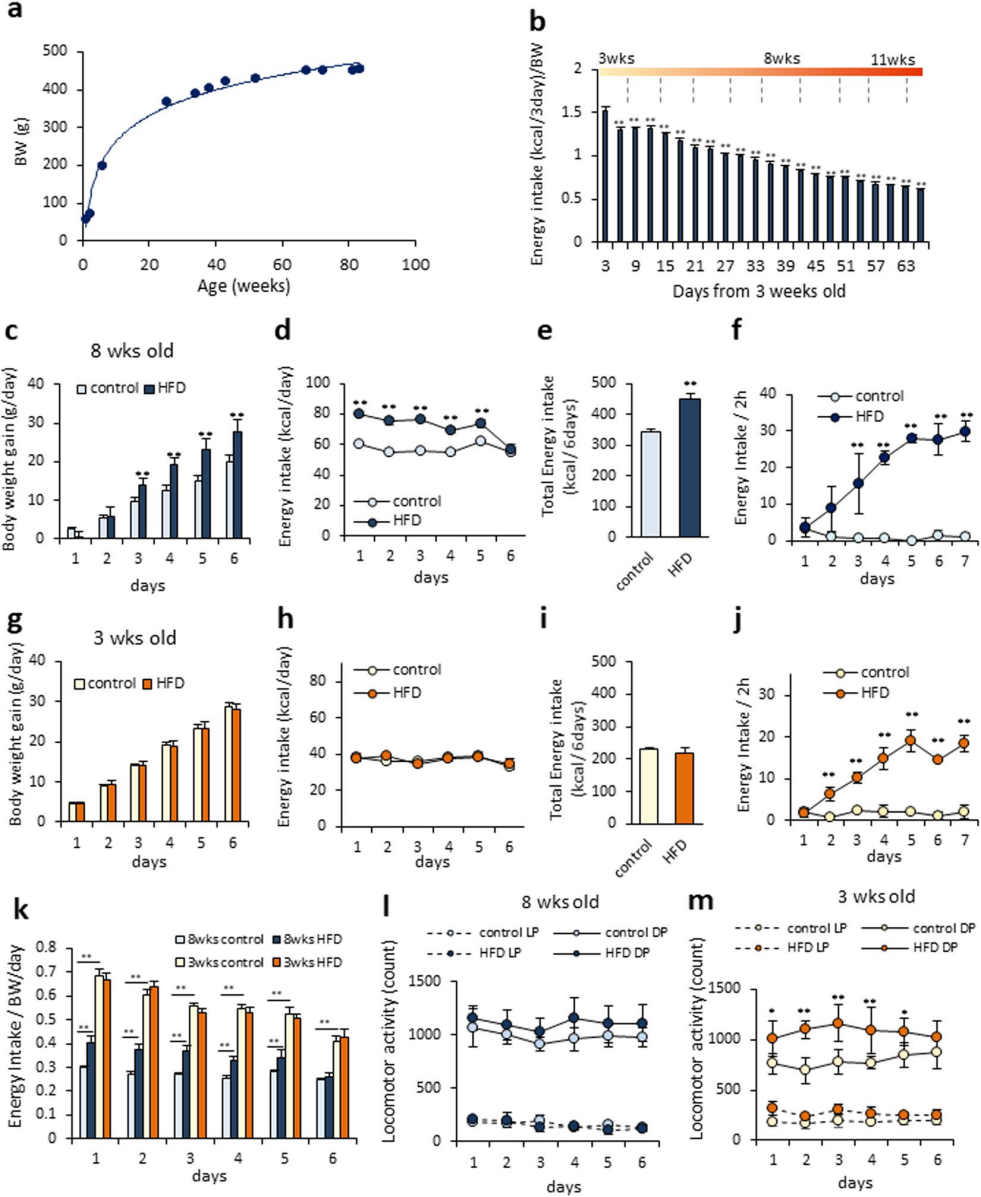
The characteristics of feeding regulation in adult and juvenile rats. (**a**) Body weight (BW) changes from three to 87 weeks old in male Wistar rats (n = 4–8). The curve was best fit with the equation: (y = 523.5–285.2/(1 + (x/28.7)^2^). (**b**) EI per BW from three to 12 weeks old in male Wistar rats (n = 4). **P < 0.01 vs EI per BW at day 3, One-way ANOVA, Tukey’s multiple range test. (**c**,**d**) BW gain (**c**) and EI (**d**) for six days under CD or HFD-fed conditions in adult rats (n = 7, 8). (**e**) Total EI for six days in adult rats (n = 8, 8). (**f**) EI for two hours for seven consecutive days in adult rats (n = 3, 3). (**g**,**h**) BW gain (**g**) and EI (**h**) for six days under CD or HFD-fed conditions in juvenile rats (n = 8, 8). (**i**) Total EI for six days in juvenile rats (n = 8, 8). (**j**) EI for two hours for seven consecutive days in juvenile rats. (**k**) EI per BW for six days in adult and juvenile rats fed a CD or HFD (n = 3, 3). (**l**,**m**) The locomotor activity of adult (**l**) and juvenile (**m**) rats fed either a CD or HFD for six consecutive days. LP indicates the light phase. DP indicates the dark phase (n = 4, 4). (**c**,**d**,**f**,**d**,**h**,**i**,**k**,**l**,**m**) **P < 0.01, Two-way ANOVA, Tukey’s multiple range test. (**e**,**i**) **P < 0.01. Unpaired t-test.

There was a rapid increase in BW after weaning, which continued to increase moderately after 40 weeks in Wistar male rats (Fig. 1a; y = 523.5–285.2/(1 + (x/28.7)^2^)). The EI per body weight decreased with growth (Fig. 1b). In eight-week-old adult rats, BW gain and EI were significantly increased under high fat diet (HFD) conditions (BW; F_1,65_ = 118.54, P < 0.01, EI; F_1,65_ = 144.17, P < 0.01, Fig. 1c) compared with a control diet (CD) (Fig. 1d,e). However, in three-week-old juvenile rats, there was no difference in BW gain or EI between the CD- and HFD-fed rats (BW; F_1,70_ = 0.135, P > 0.05, EI; F_1,70_ = 0.076, P > 0.05, Fig. 1g–i).

Our results suggest that it is possible that juvenile rats have an incomplete or no reward system compared to adult rats. In order to verify reward-related feeding regulation, HFD was given to adult and juvenile rats for only two hours each day after fasting for seven consecutive days (outside of experimental periods, all animals were fed standard chow). The EI from the HFD was significantly increased compared with EI for the CD in both the adult (F_1,24_ = 138.77, P < 0.01) and juvenile (F_1,24_ = 197.69, P < 0.01) rats (Fig. 1f,j). The maximal EIs from the HFD in the adult and juvenile rats were approximately 30 kcal and 20 kcal, respectively, which corresponds to approximately 50% of the daily EI from the CD. Thus, there were no differences in response to the highly palatable food between the adult and juvenile rats. These results indicate the presence of a reward system in juvenile rats.

Upon analyzing EI per BW for six days based on the results shown in Fig. 1c,d,g,h, the EI from the CD in the juvenile rats was more than double that of the EI from the CD in the adult rats (F_3,140_ = 420.92, P < 0.01, Fig. 1k). These data suggest that juvenile rats consume a maximum amount of EI per day, regardless of whether they are fed a CD or HFD.

Even with the same caloric intake, HFD is known to induce significantly higher BW increase compared to CD^19^. However, there was no difference in BW gain between the CD and HFD groups in the juvenile rats, despite the same caloric consumption. Locomotor activity was therefore compared between the CD- and HFD-fed adult and juvenile rats (Fig. 1l,m). In the adult rats, there were no differences in locomotor activity in both the light (LP) and dark phases (DP) between the CD and HFD groups (Fig. 1l). However, the locomotor activity of the juvenile HFD-fed rats in the DP was significantly increased compared with that of the CD-fed juvenile rats (Fig. 1m). This suggests that HFD-fed juvenile rats showed a higher energy expenditure than CD-fed juvenile rats, which may explain the reason for the similar BW gains that were observed between CD- and HFD-fed juvenile rats, even with similar caloric consumptions.

### The immaturity of GABA neurons in the ventral tegmental area in juvenile rats

**Figure 2:**
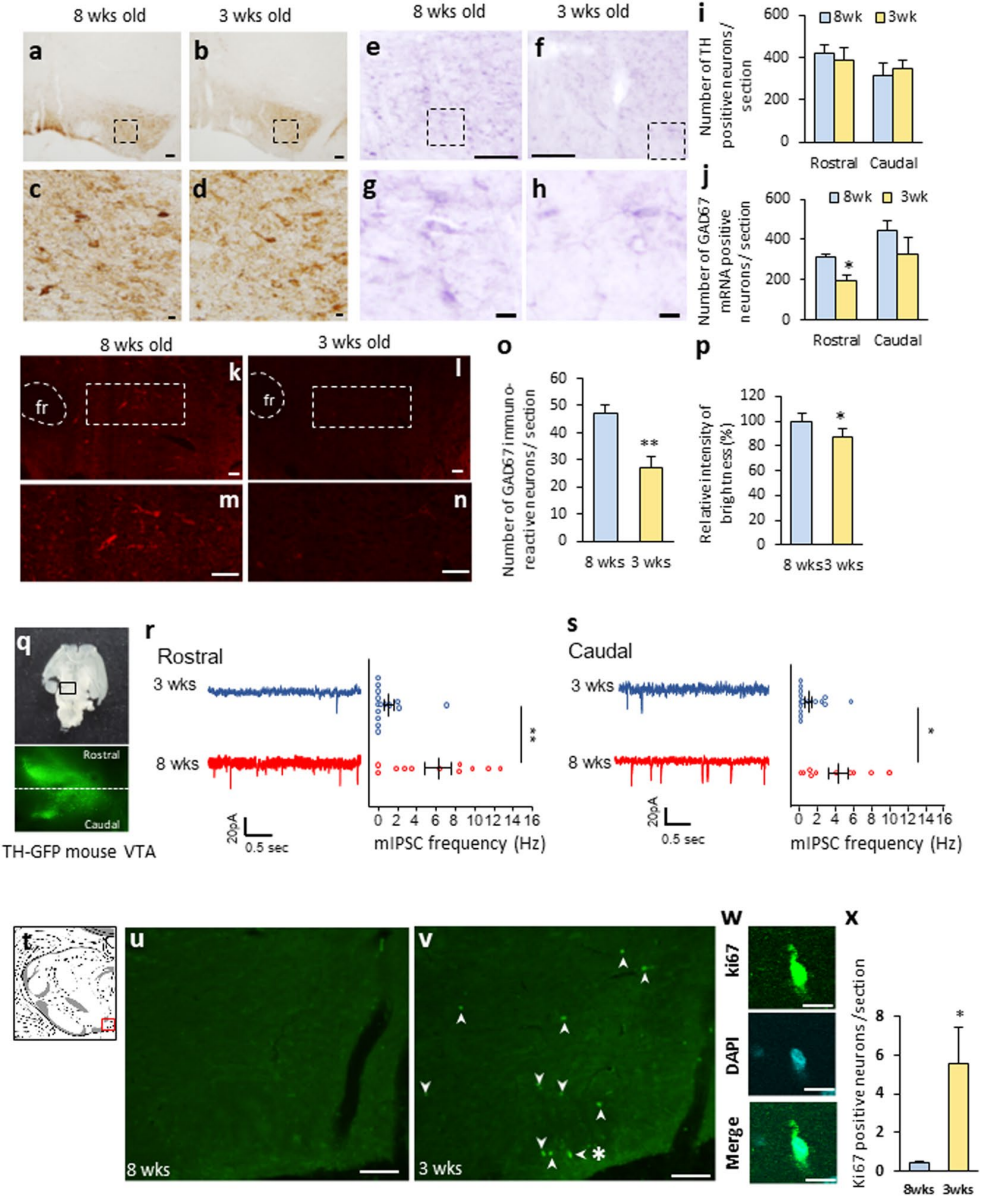
The immaturity of GABA neurons in the ventral tegmental area in juvenile rats. (**a**–**d**) The distribution of tyrosine-hydroxylase (TH) immunoreactive neurons in adult (**a**,**c**) and juvenile rats (**b**,**d**). (**c**,**d**) Enlarged image of dotted area in a and b, respectively. Scale bars in a, b = 100 μm, c, d = 10 μm. (**e**–**f**) GAD67 mRNA-positive neurons of adult (**e**,**g**) and juvenile rats (**f**,**h**). (**g**,**h**) Enlarged image of dotted area in (**e**,**f**) respectively. Scale bars in e, f = 100 μm, g, h = 10 μm. (**i**) The number of TH-positive neurons per section in the rostral and caudal VTA in adult and juvenile rats (n = 5, 5). (**j**) The number of GAD67-mRNA positive neurons in the rostral and caudal VTA in adult and juvenile rats (n = 3, 3). *P < 0.05, unpaired t-test. (**k**–**n**) Distribution of GAD67-containing neurons in the VTA in adult (**k**,**m**) and juvenile (**l**,**n**) rats. (**m**,**n**) Indicate the enlarged image of dotted area in  k and l, respectively. fr: fasciculus retroflexus. Scale bars = 50 μm. (**o**) The number of GAD67 positive neurons per VTA-containing section in adult and  juvenile rats (n = 6, 6). (**p**) The relative intensity of brightness (n = 6, 6). **P < 0.01, *P < 0.05, paired t-test. (**q**) The horizontal brain section slice of TH-GFP mouse used in a patch-clamp experiment (upper panel), and the distribution of TH-positive neurons in the VTA of TH-GFP mouse (bottom panel). The fluorescence image located on the bottom is an enlarged image of the square area of the upper panel. (**r**) Rostral region of the dopamine neurons (identified by TH-driven GFP) had reduced frequency of mIPSC (n = 15 for three weeks and n = 13 for eight weeks). (**s**) Caudal region of dopamine neurons had reduced frequency of mIPSC (n = 15 for three weeks and n = 13 for eight weeks). **P < 0.01, *P < 0.05, unpaired t-test. (**t**) Scheme of the VTA area shown in Figures (**u**,**v**). (Swanson (2004) under the conditions set forth by Creative Common BY-NC 4.0 license (http://creativecommons.org/licenses/by-nc/4.0/legalcode)). (**u**,**v**) The distribution of ki67-positive neurons in the VTA of adult (**u**) and juvenile (**v**) rats. Scale = 100 μm. Arrow heads in image v indicate ki67-positive neurons. (**w**) Enlarged image of a labelled neuron (*) in image v. The top, middle and bottom indicate ki67 staining, DAPI nuclear staining and a merged image, respectively. Scale bar = 10 μm. (**x**) The number of ki67-positive neurons in the VTA per section. *P < 0.05, unpaired t-test. (n = 3, 3).

To investigate the mechanisms whereby juvenile rats consume the maximum amount of EI per day regardless of whether they are fed a CD or HFD, brain areas which are related to reward were examined histologically. Afferent brain regions to the VTA were examined by injecting a retrograde tracer in VTA of juvenile and adult rats. However, no significant differences were observed between the rat groups as a result of this injection (Fig. S1). Furthermore, no significant differences were observed regarding projection from the VTA to the NAc between the rat groups (Fig. S2). We next focused on the distribution of dopamine and GABA neurons in the VTA. Immunostaining for tyrosine hydroxylase (TH) containing dopamine neurons showed no significant differences between the adult and juvenile rats in both the rostral and caudal regions (Fig. 2a–d,i). *In situ* hybridization for glutamic acid decarboxylase (GAD) 67 showed that the number of GAD67 mRNA-containing neurons in the juvenile rats tended to be significantly lower in the rostral region, as well as in caudal region of the VTA (Fig. 2e–h,j). In juvenile rats, there was approximately a 40% and 25% reduction in number of GAD67 mRNA-positive neurons in rostral and caudal regions, respectively, compared with the adult rats. When analyzing GAD67-containing GABA neurons using immunohistochemistry, abundant GAD67 containing neurons and fibers were found in the VTA of the adult rats (Fig. 2k,m). However, very few GAD-67 containing neurons and fibers were seen in the VTA of the juvenile rats (Fig. 2l,m). The percentage of GAD67-containing neurons in the juvenile rats was approximately 50% compared with that in the adult rats (Fig. 2o). The relative intensity of brightness of the VTA in the juvenile rats, which is considered to reflect the axonal, terminal and cell bodies of GABA neurons, was significantly lower compared with that of the adult rats (Fig. 2p).

Dopamine neurons in the VTA are known to be regulated by an inhibitory input from GABA neurons within the VTA^12,20^. Therefore, the inhibitory input in dopamine neurons in the VTA was electrophysiologically examined using brain slices from the juvenile and adult TH-GFP mice in horizontal sections (Fig. 2q). In identified VTA dopamine neurons, we found a significantly decreased frequency of miniature inhibitory post synaptic currents (mIPSC) onto dopamine neurons in the juvenile rats (rostral; 0.92 ± 0.47 Hz, caudal; 0.93 ± 0.63 Hz) compared to the adult rats in both the rostral (Fig. 2r) and caudal regions (5.95 ± 1.37 Hz, caudal; 4.26 ± 1.08 Hz) (Fig. 2s).

In order to investigate the development process of VTA neurons in the juvenile rats, ki67, a marker of neuronal proliferation, was next examined in the VTA (Fig. 2t). There were almost no ki67 positive cells in the adult rats (Fig. 2u), whereas a significant number of ki67 positive neurons were found in the VTA of the juvenile rats (Fig. 2v–x). In addition, in the juvenile rats, approximately 35% of ki67 positive neurons in the VTA were GAD67 mRNA-positive neurons (Fig. S3a,b). This was a significantly higher percentage than that of the adult rats (Fig. S3b). These results suggest that the VTA neurons in the juvenile rats, especially GABA neurons, are immature and under the process of development.

### The characteristic of feeding regulation in adolescence rats

**Figure 3:**
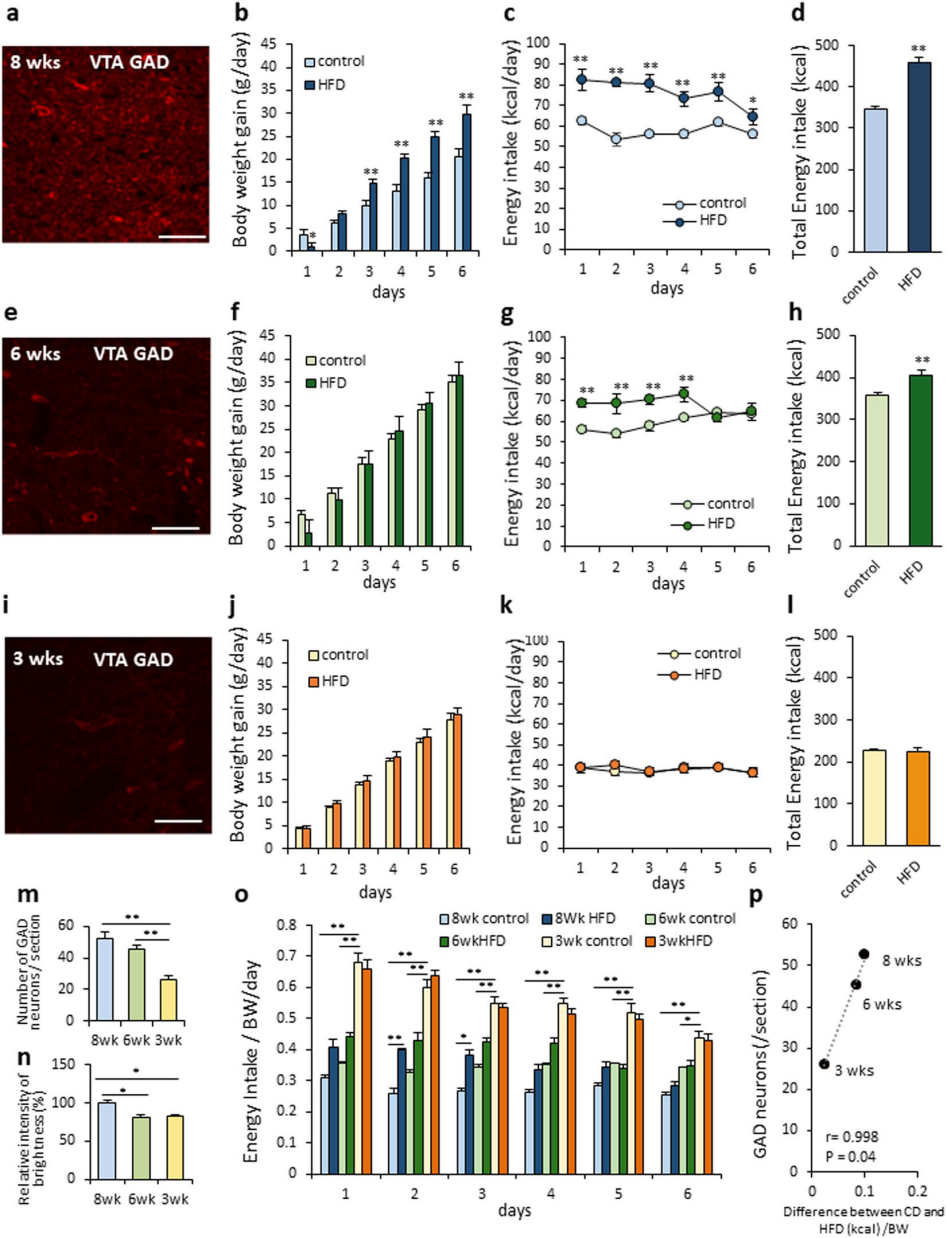
Comparison of GAD67 positive neurons, feeding and BW gain among juvenile, adolescence and adult rats. (**a**,**e**,**i**) GAD67-positive neurons in adult (**a**), adolescence (**e**) and juvenile (**i**) rats, respectively. (**b**,**f**,**j**) The BW gain under a CD- or HFD-fed condition in adult (n = 10, 9) (**b**), adolescence (n = 4, 4) (**f**), and juvenile (n = 8, 9) (**j**), respectively. **P < 0.01, Two-way ANOVA, Tukey’s multiple range test. (**c**,**g**,**k**) The EI under a CD- or HFD-fed condition in adult (**c**), adolescence (**g**), and juvenile (**k**), respectively. *P < 0.05, **P < 0.01, Two-way ANOVA, Tukey’s multiple range test. (**d**,**h**,**l**) Total EI for six days under a CD- or HFD-fed condition in adult (n = 10, 9) (**d**), adolescence (n = 4, 4) (**h**), and juvenile (n = 8, 9) old rats (**l**), respectively. **P < 0.01, unpaired t-test. (**m**,**n**) The number of GAD67-positive neurons (**m**) and the relative intensity of brightness in the adult (n = 10), adolescence (n = 5) and juvenile (n = 6) rats. *P < 0.05, **P < 0.01, One-way ANOVA, Tukey’s multiple range test. (**o**) The EI per BW for six days in the adult (n = 8, 9), adolescence (n = 4, 4) and juvenile (n = 10, 10) rats fed a CD or HFD. *P < 0.05, **P < 0.01, Two-way ANOVA, Tukey’s multiple range test. (**p**) The correlation between the number of GAD67-positive neurons and the difference of EI from CD and HFD per BW at day 1. r = 0.998, P = 0.04.

To clarify the relationship between the development of GABA neurons in the VTA and the features of feeding patterns, we compared the age-dependent effects of a HFD by comparing EI and GABA neuron development in juvenile (three weeks old), adolescent (six weeks old) and adult (eight weeks old) rats. Abundant GAD67 containing GABA neurons and fibers were confirmed in the adult rats, and following a HFD, the rats exhibited a high BW gain and EI compared to the CD-fed group (BW; F_1,85_ = 124.68, P < 0.01, EI: F_1,85_ = 153.19, P < 0.01, Fig. 3a–d). The juvenile rats showed poorly-developed GAD containing neurons (Fig. 3i), with no difference in BW gain and EI between the HFD- and CD-fed groups (BW; F_1,75_ = 2.085, P > 0.05, EI; F_1,75_ = 0.279, P > 0.05, Fig. 3j–l). Regarding the adolescent rats, as shown in Fig. 3e, the somata of the GAD67 containing neurons were found clearly, but the development of fibers was lower compared with that of the adult rats (Fig. 3e). As for the functional study, the EI from the HFD was slight, but significantly higher compared with the CD (F_1,30_ = 45.05, P < 0.01, Fig. 3g,h) in the adolescent rats. There were no differences shown in BW gain between the CD- and HFD-fed rats (F_1,30_ = 0.0026, P > 0.05, Fig. 3f).

Comparing the histological features of VTA GABA neurons among the juvenile, adolescent and adult rats, the number of GAD67-containing neurons increased age-dependently from three to eight weeks old (Fig. 3m). The relative intensity of brightness in the VTA was higher in the adult rats compared with the adolescence and juvenile rats (Fig. 3n). When analyzing EI per BW among the three different age groups, that from a CD was larger in younger rats (F_5,190_ = 128.63, P < 0.01, Fig. 3o). Because there is a clear age-dependent correlation between differences in the EI from a CD and HFD per BW, and the number of GAD67 containing neurons (r = 0.998, P < 0.05, Fig. 3p), it can be considered that the development of GABA neurons in the VTA contribute to the difference of EI between less palatable (CD) and high palatable (HFD) food in an ad lib condition.

### The deletion of VTA GABA neurons increased EI from CD

**Figure 4:**
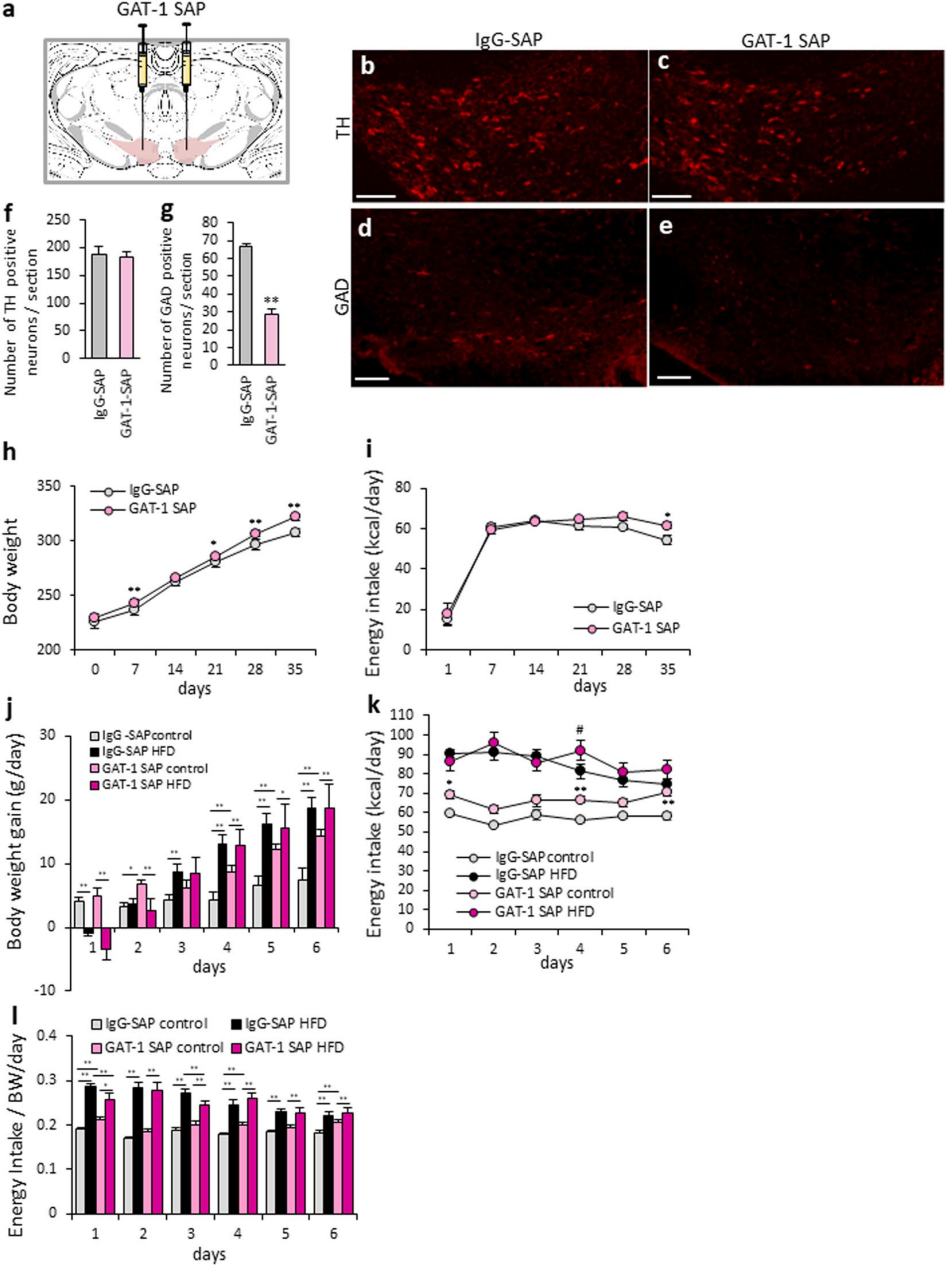
The deletion of GABA neurons in the VTA of adult rats. (**a**) Scheme of the GAT-1-injected area. (Swanson (2004) under the conditions set forth by Creative Common BY-NC 4.0 license (http://creativecommons.org/licenses/by-nc/4.0/legalcode)). (**b**,**c**) TH immunostaining in the VTA at 50 days from control IgG-SAP (**b**) or GAT-1 SAP (**c**) injection. (**d**,**e**) GAD67 immunostaining in the VTA at 50 days from control IgG-SAP (**d**) or GAT-1 SAP (**e**) injection. Scale bars = 50 μm. (**f**) The number of TH-positive neurons per section. (n = 7, 6). (**g**) The number of GAD67-positive neurons per section. P < 0.01, unpaired t-test. (n = 7, 6). (**h**,**i**) The BW (**h**) and EI (**i**) after IgG-SAP or GAT-1 SAP injection into the VTA. *P < 0.05, **P < 0.01. Two-way ANOVA, Tukey’s multiple range test. (n = 7, 6). (**j**) BW change under a CD- or HFD-fed condition in IgG-SAP or GAT-1 SAP injected rats. *P < 0.05, **P < 0.01. Two-way ANOVA, Tukey’s multiple range test. (n = 6–7). (**k**) The EI under a CD- or HFD-fed condition in IgG-SAP or GAT-1 SAP injected rats. (n = 6–7) *P < 0.05, **P < 0.01. vs. IgG-SAP control group. ^#^P < 0.05. vs. IgG-SAP HFD group. Two-way ANOVA, Tukey’s multiple range test. (**l**) EI per BW for six days in IgG-SAP or GAT-1 SAP-injected rats fed a CD or HFD. (n = 6–7) *P < 0.05, **P < 0.01, Two-way ANOVA, Tukey’s multiple range test.

In order to further confirm the physiological function of GABA neurons in the VTA, we selectively deleted GABA neurons in eight-week-old adult rats by using a neurotoxin. GABA transporter-1 (GAT-1) saporin (SAP) was bilaterally injected into the VTA in eight-week-old adult rats (Fig. 4a). Seven weeks after injection, we confirmed a significant reduction of GAD67-containing neurons in GAT-1 SAP injected rats, compared to the control IgG-SAP-injected rats (Fig. 4d,e,g). However, there were no significant differences in the distribution and number of TH-containing dopamine neurons (Fig. 4b,c,f).

When monitoring BW after injection under standard chow- (CE2) fed conditions for 35 days, the BW of the GAT-1 SAP-injected group was slightly, but significantly, increased compared with that of IgG-SAP-injected rats (F_1,55_ = 59.63, P < 0.01, Fig. 4h). The EI was also significantly increased in the GAT-1 SAP-injected rats (F_1,55_ = 4.48, P < 0.05, Fig. 4i).

Under HFD-fed conditions, the EI from the HFD in the IgG-SAP- and the GAT-1 SAP-injected groups showed almost no difference (Fig. 4k). However, under CD-fed conditions, the EI was significantly increased in the GAT-1 SAP-injected group compared to the control IgG-SAP-injected group (F_3,110_ = 229.11, P < 0.01, Fig. 4k). In addition, BW gain in the IgG-SAP-injected group was dramatically increased under HFD-fed conditions (F_3,110_ = 40.53, P < 0.01, Fig. 4j). BW gain in the GAT-1 SAP-injected group was also slightly, but significantly, increased in the HFD-fed group compared with the CD-fed group (Fig. 4j). Comparisons within the CD-fed group, BW gain was significantly increased in the GAT-1 SAP-injected group, compared with the IgG-SAP-injected group (Fig. 4j).

Under the CD-fed conditions, the EI per BW in the GAT-1 SAP-injected rats was also significantly higher compared with that of the IgG-SAP-injected rats (F_3,110_ = 178.91, P < 0.01, Fig. 4l).

## Discussion

During the neonatal and juvenile periods in which animals are in the developing stage, high energy intake is required^1–3^; thus, juvenile animals are expected to eat more than adults, i.e. EI/BW is much higher in juvenile animals compared to adults. In general, a HFD stimulates the reward-related brain regions and induces reward-related consumption^21^; therefore, EI from a HFD is higher than that from normal chow (less palatable) food in adults. In the current study, as shown in Fig. 1c–f, the adult rats fed with a HFD showed increased EI, which led to BW gain. However, we also found that, in the juvenile rats, the EI from the HFD and that from the CD were of a similar amount. In addition, BW gain in the juvenile rats was not increased even under HFD feeding.

This phenomenon was also reported by Tinkov *et al*., but its underlying mechanism was not clarified^22^. However, our present data, taken together with by Tinkov *et al*.’s findings, indicate the involvement of a reward system for this phenomenon, which may be caused by the similar EI from a CD and HFD in weaning rats.

Therefore, in order to examine whether the reward system is functional at a juvenile stage, the juvenile rats were fed with either a CD or HFD for only two hours, for seven consecutive days. Surprisingly, these rats responded to the high palatable food (HFD), indicating that the reward system is fully functional in juvenile rats. It can be said from these results that juvenile rats consume the maximum amount of EI per day, regardless of whether they are fed a CD or HFD.

A HFD is known to significantly increase BW even if the calorie intake is similar to that of a CD^19^. However, in the present study, no difference was observed regarding BW gain between the CD and HFD groups in the juvenile rats, regardless of the caloric intake being the same (Fig. 1g,h). On the other hand, locomotor activities were significantly increased only in the HFD-fed juvenile rats. It can be speculated that hyperactivity in the HFD-fed juvenile rats may promote energy expenditure, and have contributed to the similar weight gains as those in the CD-fed rats in the current study.

During the neonatal period, animals, including humans, need to maximize their caloric intake in order to maintain appropriate metabolic responses to ensure growth and survival^23^. The promotion of EI in juveniles may be supported by the fact that the anorexigenic peptide leptin does not inhibit food intake or energy expenditure during the first two to three postnatal weeks^24–26^. The immaturity of the hypothalamic circuit seems to contraindicate a role for the hypothalamus in relaying leptin’s action on feeding and energy balance in neonatal and juvenile animals.

In the present study, we demonstrated for the first time that maximizing caloric intake during neonatal and juvenile periods is also promoted by the immaturity of the limbic system, as well as the hypothalamus. The results of our histological and electrolophysiological analyses clearly indicate the immaturity of the VTA GABA neurons in juvenile rats.

The projection target of VTA dopamine neurons, such as NAc, may promote reward-related behavior^9,27^. As for the regulatory mechanism for VTA dopamine neuron activity, Zessen *et al*. reported that the VTA GABA neurons directly suppress the activity and excitability of neighboring dopamine neurons, which ultimately regulate the overall reward consumption^12^. The present study clearly showed that, in three-week-old juvenile rats, neurons such as GABA neurons are still under proliferation in the VTA with less inhibitory input into the dopamine neurons. Our results are consistent with those of the previous study, which reported that the dopamine neurons in the VTA fire faster in adolescent rats (5–7 weeks old) than in adult rats (12–15 weeks old)^18^. It is thus rational to consider that GABA neurons in the VTA are still immature and have little inhibitory input to dopamine neurons in juvenile rats, and therefore lead to promote activation of dopamine neurons.

There are two kinds of VTA GABA neurons; those that contribute to modulating the activity of local dopamine neurons, which is considered to be related to reward or aversion action^28^, and those with long-range GABA projections from the VTA to the NAc, which modulate associative learning^28^. In the present study, we were unable to distinguish these two types of VTA GABA neurons; however, since only a small number of VTA GABA neurons are known to project to the NAc^20^, our data may mainly reflect the function of VTA GABA neurons, which is projection within the VTA. Supporting this idea, the number of GAD67 mRNA-positive neurons was lower in both the rostral and caudal regions of the VTA in the juvenile rats (Fig. 2j), in which there were also similar inhibitory inputs into the tyrosine hydroxylase (TH) neurons in both regions (Fig. 2r,s). These results suggest that immaturity of the GABA neurons is observed throughout the VTA, and is related to the regulation of activity in TH-positive neurons within VTA.

Recent studies revealed the physiological roles of VTA GABA neurons, such as regulating drive conditioned place aversion^20^. It has been reported that VTA GABA neuron activity attenuates the ability of cue to trigger reward-seeking behavior^29^, and the activation disrupts reward consumption^12^. The present study showed that juvenile rats, which have immature and non-functional GABA neurons, consume the maximum amount of EI per a day, regardless of a CD or HFD. These results correspond well to those of reports by Wakabayashi *et al*.^29^ and Zessen *et al*.^12^. However, we also showed that within a short time (two hours), consumption of a HFD in juvenile rats was similar to that of adult rats. Thus, immaturity of GABA neurons in the VTA may be functionally related to the promotion of EI for longer periods, which is promotion of EI in daily basis, but not in hourly basis.

In addition to the reward-related function, VTA GABA neurons regulate locomotor activity_,_ which is spontaneously increased by the deletion of these neurons^30^. Moreover, the VTA-NAc dopamine pathway plays a critical role in locomotor response to drugs^31–33^. Taking into consideration the hyperactivity observed in the HFD-fed juvenile rats in this study, it is possible to suggest that dopamine neurons are easily activated in juvenile rats due to the immaturity of the GABA neurons. Increased locomotor activity in the HFD-fed juvenile rats may reflect increased activity of dopamine neurons. Further studies are required to clarify the effect of HFD on locomotor activity in juvenile rats.

Our study clearly showed that the degree of development of VTA GABA neurons is related to the amount of CD and HFD consumption, and BW gain (Fig. 3). Interestingly, we found that the number of GAD-positive neurons and differences in EI from a CD and HFD had a significant correlation. This indicates that the age-dependent development of VTA GABA neurons is a key factor that generates proper reward consumption in adult rats. We also revealed that the deletion of VTA GABA neurons in the adult rats increased CD consumption close to the level of HFD consumption, which was similar to the effect observed in the juvenile and adolescent rats. It is possible to hypothesize that because VTA GABA neurons are still immature in juvenile rats, these rats are not capable to distinguish high and less palatable foods, and therefore consume a CD and HFD to the same extent in ad lib conditions. This may lead to the promotion of EI in the newborn and juvenile stages that support the high energy requirement in growth period. However, in the present study, the difference in EI from a CD and HFD was not completely abolished, regardless 50% decrease of GAD positive neurons from VTA after injection of GAT-1 SAP in adult rats. This might be explained by the compensatory mechanism in adult rats after development of the reward-related neural circuit. Further studies to clarify this contradiction are needed in future.

In conclusion, this is the first study to report the characteristics of feeding regulation and immaturity of VTA GABA neurons in juvenile rats. The physiological significance of the immaturity of VTA GABA neurons is to increase EI regardless of high or less palatable food, and yet achieve efficient growth.

From a clinical point of view, at least 35% of children younger than five years old are diagnosed as overweight or obese in the USA^34^. Several gene mutations, such as in melanocortin type 4 receptors, are reported to be the cause of early onset of obesity^35^. In addition, our present data indicate that any factor, including environmental or genetic, that interferes with the development of VTA GABA neurons, may ultimately lead to the development of early onset obesity.

## Methods

### Animals and housing

Male Wistar rats (three weeks old was classified juvenile; six weeks old was classified as adolescent; and eight weeks old was classified as adult) were purchased from Japan SLC. Tyrosine hydroxylase (TH) green fluorescence protein expression transgenic (TH-GFP) mice were obtained from the Institute of Physical and Chemical Research^36,37^. Juvenile male mice and adult male mice were used for electrophysiological experiments. All TH-GFP mice used in this study were genetically heterozygous. The animals were kept on a 12-h light/dark cycle and given conventional food (CE-2; Clea, Osaka, Japan) and water *ad libitum* in individual cages. The lights were turned on at 07:00 am and turned off at 19:00. All experimental procedure and care of animals were carried out according to relevant guidelines and regulations and approved by Fukushima Medical University Institute of Animal Care and Use Committee.

### The analysis of BW change in male Wistar rats

The BW of male Wistar rats fed with conventional food (CE-2) at 3–16, 26, 35, 39, 44, 52, 67, 72, 83 and 85 weeks old was measured. Each measurement was made at 17:00.

### The analysis of energy intake per BW

The BW and food intake (CE-2) of male Wistar rats were measured every three days from age 3 weeks to age 12 weeks. Each measurement of BW and food intake during this period was performed at 17:00. The energy content of CE2 is 339.1 kcal/100 g. Energy intake was calculated by the amount of food intake (g) ×3.391 (kcal).

### Comparative study of BW gain and food intake among juvenile, adolescent and adult rats

Juvenile, adolescent and adult rats were fed standard chow (CE-2: Clea, Osaka, Japan), and divided into two groups. The diet of the first group was switched from standard chow to a CD (CE-7: Clea, Osaka Japan), and the diet of the second group was switched from standard chow to a HFD (HFD32, Clea, Osaka, Japan). BW and food intake were measured at 17:00 for six consecutive days. The % kcal from the total energy of each diet was as follows: CE-2, protein 29%, fat 12.8%; CE-7: protein 20.4%, fat 10.4%; and HFD, protein 20.9%, fat 56.7%. The energy contents of the CE-7 and HFD were 337.1 kcal/100 g and 507.6 kcal/100 g, respectively. The energy intake was calculated from these energy contents.

### Examination of preference for high palatable food

Juvenile and adult rats fed with standard chow were divided into two groups. At the day of the experiment, food was ceased at 11:00 and the control diet, CE-7, or HFD was provided at 13:00 for two hours, and the amount of food intake was measured at 15:00. Thereafter, standard chow was given. This protocol was repeated for seven days.

### Measurement of locomotor activity

Locomotor activity was measured using an activity monitoring system (ACTIMO-100: Shinfactory, Fukuoka, Japan), which estimates locomotor activity by the count of rat interference with an infrared beam radiated in X and Y directions inside the cage. Juvenile and adult rats were habituated in individual measurement cages for 24 h on a standard diet, CE2. At the onset of the experiment, food was switched to either a CD (CE7) or HFD, and food intake and locomotor activity was measured for six days. During this experimental period, BW was measured once a day at 17:00.

### Immunostaining for tyrosine hydroxylase (TH)

Juvenile and adult rats fed with a standard diet, CE-2, were injected with an overdose of anesthesia and perfused intracardially with 4% paraformaldehyde (PFA), and 0.2% picric acid.

Serial coronal sections (40 μm) from the ventral tegmental area (VTA) (−4.9 mm to −5.8 mm from the bregma) were collected from each rat using a freezing microtome. The VTA-containing sections were washed in PBS (0.01 M, pH7.4), then incubated for 20 min with 0.3% H_2_O_2_. Next, the sections were incubated for 1 hour in a blocking solution comprising of 0.3% TritonX-100, 2% bovine serum albumin (BSA), and 2% normal goat serum (NGS) and then incubated with mouse monoclonal anti-TH antibody (22941, 1: 2000, IMMUNOSTAR, WI) in the blocking solution overnight at 4 °C. The sections were then incubated with biotinylated goat anti-mouse IgG (diluted to 1:500, Vector Laboratories, CA) for 30 min, followed by incubation with an avidin-biotin-peroxidase complex (Vectastain Elite ABC Kit; Vector Laboratories, CA) for 60 min. Immunoreactions were visualized by incubation in a 0.02% diaminobenzidine solution containing 0.015% H_2_O_2_ for 5 min. After color development, the sections were mounted on glass slides and covered. The number of TH positive neurons was counted under a microscope. We analyzed the TH-positive neurons in two areas of the VTA (rostral and caudal regions) based on a report by Olson *et al*.^38,39^. The rostral region was −4.9 to −5.4 mm from the bregma. The caudal region was −5.5 to −5.8 mm from the bregma. As a point of reference, the caudal regions contain interpeduncular nucleus (IP), whereas the rostral regions do not.

### *In situ* hybridization for GAD67

Sense and antisense rGAD67 riboprobes were designed based on a previous article^40^. rGAD67 was incorporated into the multicloning site at the pGEM-T Easy vector, and the sense and antisense probes were synthesized with T7 and SP6 RNA polymerases, respectively, by using the digoxigenin (DIG) RNA labeling kit (Roche Diagnostics, Boehringer Mannheim, Germany). The sequence of the antisense riboprobe was as follows:

CCATCTCAAACTCTTCTCTGTTTTTAATCTTGGCATAGAGGTATTCAGCCAGCTCCAGGC.

*In situ* hybridization for GAD67 was performed in a free-floating method. Juvenile and adult rats fed a standard diet, CE-2, were perfused in the same manner as that described above. VTA containing sections (−4.9 mm to −5.8 mm from the bregma) were washed in DEPC-PBS, and then incubated in 1.5% H_2_O_2_ in DEPC-PBS for 30 min to inhibit endogenous peroxidase activity. The sections were incubated in 0.2 N HCl for 20 min, and then incubated in proK (1 μg/ml) in prok buffer (0.1 M EDTA in 0.2 M Tris-HCl [pH 8.0]) for 15 min at 37 °C, and washed in DEPC-PBS. Next, the sections were acetylated in 0.25% acetic anhydride in 0.1 M triethanolamine-HCl (pH 8.0) for 10 min, and were prehybridized in hybridization buffer containing 1 × SSC, 50 μg/ml heparin, 0.001 M EDTA (pH 8.0), 50% formamide, 1 × Denhardt’s solution, 0.1% tween-20, 0.25 mg/ml yeast tRNA, 10% dextran sulfate. During prehybridization, another set of hybridization buffers was incubated at 85 °C for 10 min, and the sense or antisense probes (10–20 ng/ml) were added and denatured in the hybridization buffer at 85 °C for 3 min, then put on ice for 3 min. Following prehybrizidation, the sections were hybridized via incubation in a prepared probe containing hybridization buffer at 60 °C overnight.

Post-hybridization washes were performed sequentially 3 × 15 min at 50 °C in 50% formamide/2 × SSC, 2 × 15 min at 50 °C in 2 × SSC and 2 × 15 min at 50 °C in 0.2 × SSC and wash buffer (0.15 M NaCl in 0.1 M Tris-HCl [pH 7.5] [TBS] containing 0.05% Tween-20). Then, the sections were incubated for 60 min in blocking buffer (2% blocking reagent [Roche Applied Science, Upper Bavaria, Germany] in TBS). After blocking, the sections were incubated with mouse monoclonal anti-DIG antibody (11333062910, 1:400, SIGMA-ALDRICH, MO) in blocking buffer for 60 min. The sections were next washed in 0.05 M Tris-HCl buffer (pH 7.5), and incubated with biotinylated goat anti-mouse IgG (BA-9200, 1:400, VECTOR Laboratories Inc. CA) for 40 min. After that, the sections were washed in 0.05 M Tris-HCl buffer (pH 7.5), and incubated with a avidin-biotin complex (ABC kit; Vector Laboratories Inc., CA). Following washing of the sections, immunoreactions were visualized via incubation in a diaminobenzidine (DAB) solution containing nickel ammonium, and rinsed with Tris-HCl buffer. The sections were mounted on glass slide and coverslipped using Entellan new (Merk, Darmstadt, Germany). GAD67 mRNA-positive neurons in the VTA of the rostral and caudal regions were counted under a light microscope. Controls with the sense probe were run to ensure the absence of nonspecific labelling.

### Immunostaining for glutamic acid decarboxylase (GAD67)

Juvenile, adolescent and adult rats fed a standard diet, CE-2, were perfused in the same manner as described above. The VTA-containing sections (−4.9 mm to −5.8 mm from the bregma) were washed in PBS incubated with a blocking solution (0.1% Triton-X, 0.2% BSA, 0.2% NGS) for 30 min. These sections were then incubated with a mouse monoclonal anti-GAD67 antibody (MAB5406, 1:500, Millipore, CA) overnight at 4 °C. Then, the sections were incubated with Daylight 594-labelled anti-mouse IgG (ThermoFisher, IL) for 30 min, mounted with mount medium on glass slides, and covered with a cover glass. The images were acquired using a confocal laser-scanning microscope (Fluoreview FV10i; Olympus, Osaka, Japan). All images were captures in the same light intensity. GAD-positive neurons were counted from the microscope images and the relative intensity of brightness was measured by NIH image software (Image J 1.48 v, National Institute of Health).

### Electrophysiology

Whole-cell recordings were made using an EPC 800 patch clamp amplifier (HEKA) with filtering at 1 KHz using 4–6 MΩ electrodes. Horizontal brain slices (Bregma −8.10 mm to −7.6 mm) (250 μm) were prepared in an ice-cold solution containing (in mM) 230 sucrose, 2 KCl, 1 KH_2_PO_4_, 0.5 CaCl_2_, 1 MgCl_2_, 26 NaHCO_3_, and 10 D-glucose. The slices were recovered in artificial cerebral fluid (aCSF), containing (in mM) 126 NaCl 2.5 KCl, 1.2 MgCl_2_, 2.4 CaCl_2_, 1.2 NaH_2_PO_4_, 21.4 NaHCO_3_, and 10 D-glucose with 95% O_2_ and 5% CO_2_ mixed gas. Patch electrodes were filled with an internal solution containing (in mM) 120 K-gluconate, 10 KCl, 10 HEPES, 5 EGTA, 0.3 CaCl_2_, 1 MgCl_2_, 2 Mg-ATP, and 1 Na-GTP at pH 7.3 adjusted with KOH. The brain slices were transferred to a recording chamber and continuously perfused at 2–4 ml/min with gassed aCSF. Whole-cell patch recordings were performed for measurements of postsynaptic currents (PSCs), cells were voltage clamped at −60 mV, and currents were continuously recorded for 5–10 min in each test solution (IPSCs were measured in the presence of 20 μM 6-cyano-7-nitroquinoxaline-2,3-dione disodium salt [CNQX] and 50 μM D(−)-2-amino-5-phosphonopentanoic acid [AP5]. The frequency of PSCs was calculated before and after the application of experimental solutions from recordings of 60 seconds in each solution. Data from rostral and caudal regions of the VTA were analyzed using the Clampfit software (Molecular devices).

### Immunostaining for ki67

Juvenile and adult rats fed a standard diet, CE-2, were perfused in the same manner as described above. VTA containing sections (−4.9 mm to −5.8 mm from the bregma) were washed in PBS incubated with the blocking solution (0.1% triton-X, 2% BSA, 2% NGS) for 30 min. These sections were then incubated with a rabbit anti-ki67 monoclonal antibody (MA5-14520, 1:50, ThermoFisher, IL) overnight at 4 °C. Then, the sections were incubated with Alexa 488-labelled anti-rabbit IgG (ThermoFisher, IL) for 30 min and mounted with DAPI containing mount medium on glass slides and covered with a cover glass. The images were acquired using a confocal laser-scanning microscope (Fluoreview FV10i; Olympus, Osaka, Japan). Ki67 positive neurons were counted under the microscope.

### The injection of GABA transporter-1 (GAT-1) saporin (SAP) into VTA

GAT-1 SAP was purchased from the Advanced Targeting System (CA), and control IgG saporin (IgG-SAP) was used for control injections. Injections of 0.025 μg/0.5 μl GAT-1 SAP or 0.025 μg/0.5 μl IgG-SAP were administered into the bilateral VTA (5.2 mm caudal to the bregma, 0.8 mm lateral from the midline, and 8.0 mm below the surface of the skull; injection speed: 0.1 μl/min) by using modified glass pipettes under anesthesia in eight-week-old rats.

The doses of GAT-1 SAP were determined by the preliminary experiment. We tried four doses of GAT-1 SAP, 0.1, 0.05, 0.025 and 0.01 μg/0.5 μl, and 0.025 μg/0.5 μl GAT-1 SAP worked most efficiently, and non-toxically, for deletion of GABA neurons.

In order to prevent the IgG-SAP or GAT-1 SAP solution from adhering to the outside of the target area, the tip of the glass pipette was fixed to the injection site for 10 mins after injection. This conjugate, which binds to GAT-1, is internalized and causes cell death by disrupting ribosomal function^41^. The injected animals were fed standard chow, CE2. BW and food intake of the animals injected with IgG-SAP or GAT-1 SAP were measured at 17:00 for 37 consecutive days. At 38 days after injection, the animals were fed CD or HFD for six days.

### Statistical analysis

All data is presented as mean ± SEM. Student’s t-test was used for two-group comparisons. The statistical analysis of the daily changes of BW and EI, EI per BW, were analyzed by two-way or one-way ANOVA followed by Tukey’s multiple range test. All statistical tests were two-tailed, with values of 0.05 being considered statistically significant.

## Supplementary information

Supplementary Information
