# Supplementary material for: The undeveloped properties of GABA neurons in the ventral tegmental area promote energy intake for growth in juvenile rats
Source: Sci Rep. 2019 Aug 14;9:11848. doi: 10.1038/s41598-019-48336-5 (PMC6694191; doi:10.1038/s41598-019-48336-5)
Supplement: Supplementary file 1 — Supplementary Information [file 41598_2019_48336_MOESM1_ESM.pdf]

Supplementary Information

The undeveloped properties of GABA neurons in the ventral tegmental area promote energy intake for growth in juvenile rats

Yuko Maejima, Shoko Yokota, Shoichiro Horita, Kenju Shimomura

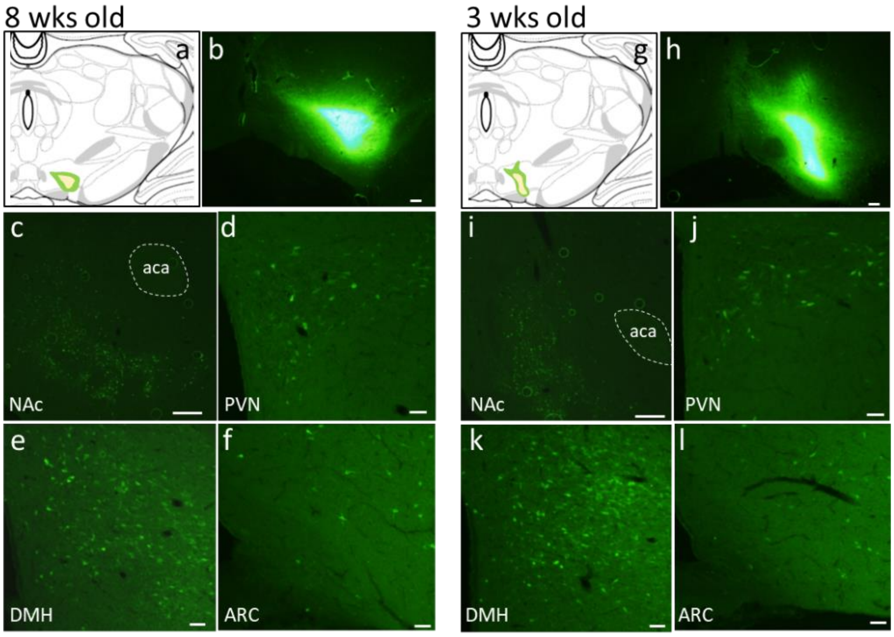

m

	8 wks	ratio (L : R)	3 wks	ratio (L : R)
PFC	++	1 : 9	+++	1 : 9
NAc	+++	0 : 10	+++	0 : 10
LSA	++ / +++	0 : 10	++/+++	0 : 10
BNST	+/++	0 : 10	+	0 : 10
MPOA	++	1 : 9	+++	1 : 9
AHA	+++	1 : 9	+++	1 : 9
PVN	+ / +++	2 : 8	+ / +++	1 : 9
SON	–	–	–	–
Hb	– / ++	1 : 9	++ / +++	1 : 9
ARC	+	0 : 10	– /+	1 : 9
VMH	– / +	1 : 9	– / +	1 : 9
DMH	++ / +++	1 : 9	+++	1 : 9
MeA	– / +	0 : 10	+ / ++	1 : 9
CGA	+ / ++	5 : 5	+ / ++	5 : 5
PBN	++	4 : 6	++ / +++	5 : 5
NTS	– / +	0 : 10	– / +	1 : 9

Supplementary Figure 1. Afferent brain regions to the VTA in adult and juvenile rats.
a, g: Scheme of the chorela toxin subunit B (CTB)-injected site in eight- (a) and three-week-old juvenile (b) rats, respectively. Bregma -5.20 mm (Swanson (2004) under the conditions set forth by Creative Common BY-NC 4.0 license (http://creativecommons.org/licenses/by-nc/4.0/legalcode)). **b, h**: Photomicrograph of the CTB injection site in adult (b) and juvenile (b) rats. Scale = 100 μm. **c-l**: Representative distribution of CTB labelled neurons in NAc (c, i) Scale = 500 μm, PVN (d, j), DMH (e, k) and ARC. Scale = 100 μm. **m**: A table of the afferent brain regions to the VTA and ratio of CTB-labelled neurons on the right and left of each nucleus. CTB Alexa Fluor 488

(Invitrogen, CA), 0.5 μl of 0.5 mg/ml, was acutely injected into the right side of the VTA (eight weeks: 5.1 mm caudal to the bregma, 0.8 mm lateral from the midline and 8.0 mm below the surface of the skull, three weeks: 4.7 mm rostral to the bregma, 0.7 mm lateral from the midline and 7.4 mm below the surface of the skull) by using modified glass pipettes under anesthesia. The injection speed was 0.5 μl / 5 min. The tip of glass pipette was fixed in the injection site for 10 mins after the injection to prevent the CTB solution from diffusing outside the target area. Six days after the administration of CTB, the animals were perfused. The brain sections were made by cutting in a freezing microtome. Injection accuracy was confirmed histologically. The densities of the CTB labelled neurons in injection site (right side) were subjectively determined by visual inspection of the brain sections as follows: +++, high; ++, moderate; +, low and -, absent. This data is inherently qualitative. (n =3, 3). L: left side, R: right side.

Abbreviations, aca: anterior commissure, anterior part, AHA: anterior hypothalamic area, ARC: arcuate nucleus, BNST: bed nucleus of the stria terminalis, CGA: central gray, alpha part, DMH: dorsomedial hypothalamic nucleus, Hb: habenular, LSA: lateral septal area, MeA: medial amygdala, MPOA: medial preoptic area, NAc: nucleus accumbens NTS: nucleus tractus slitarius, PBN: parabrahial nucleus, PFC: prefrontal cortex, PVN: paraventricular hypothalamic nucleus, SON: supraoptic nucleus, VMH: ventromedial hypothalamic nucleus

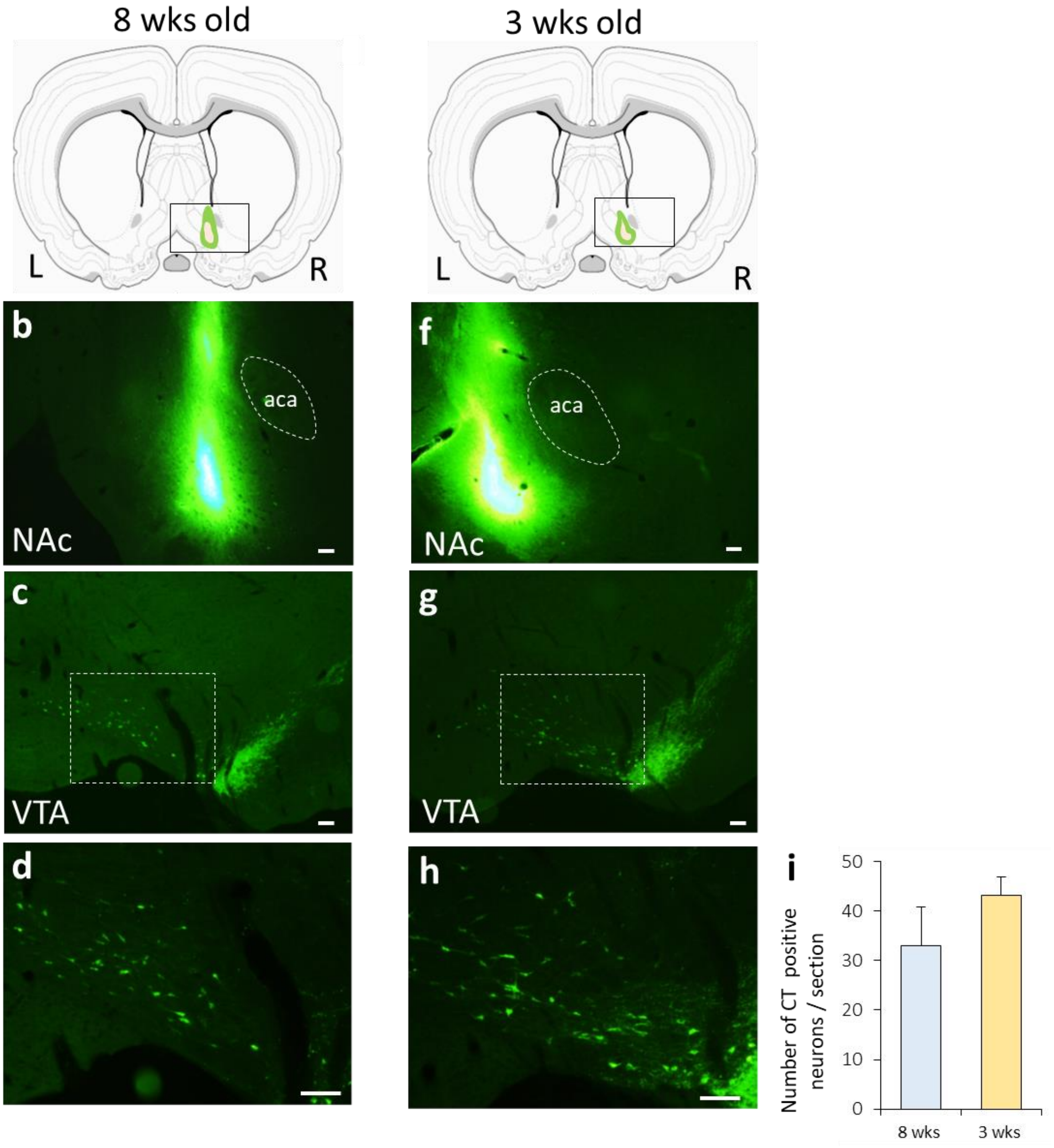

Supplementary Figure 2. Projection from the VTA to the NAc in adult and juvenile rats

a, e: Scheme of the chorela toxin subunit B (CTB)-injected site in eight-week-old adult (a) and three-week-old juvenile (e) rats, respectively. Bregma +1.00 mm (Swanson (2004) under the conditions set forth by Creative Common BY-NC 4.0 license (http://creativecommons.org/licenses/by-nc/4.0/legalcode)). L: left, R: right. **b, f**: Photomicrograph of the CTB injection site in adult (b) and juvenile (f) rats. Scale = 100 µm. **c, g**: Representative distribution of CTB-labelled neurons in adult (c) and juvenile (g) rats in the VTA. Scale 100 µm. **d, h**: Enlarged images of the dotted square in c and d, respectively. Scale 100 µm. **i**: Number of CTB-labelled neurons per section of the VTA. CTB Alexa Fluor 488, 0.5 µl of 0.5 mg/ml, was acutely injected into the right side of NAc

(eight weeks: 5.1 mm caudal to the bregma, 0.8 mm lateral from the midline and 8.0 mm below the surface of the skull, three weeks: 4.7 mm rostral to the bregma, 0.7 mm lateral from the midline and 7.4 mm below the surface of the skull). Injection of CTB was performed in the same manner as that shown in Supplementary Figure 1. aca: anterior commissure, anterior part.

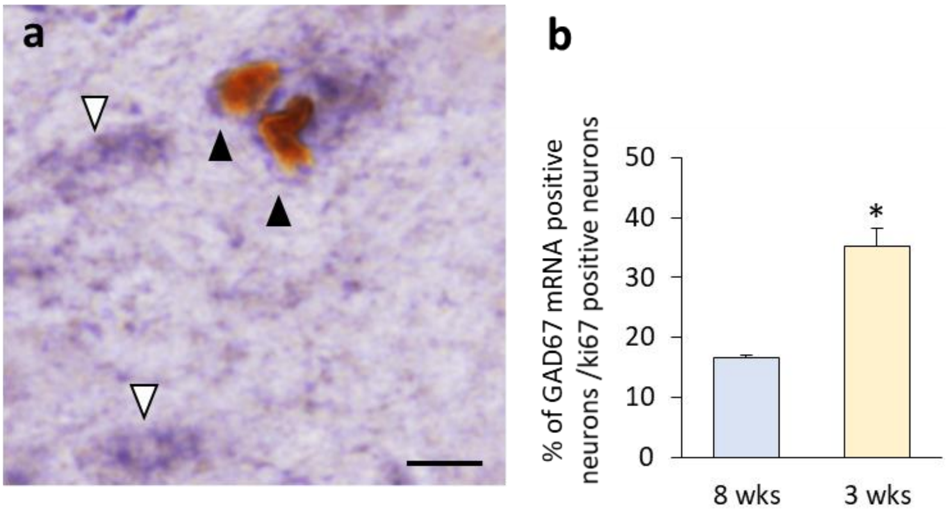

Supplementary Figure 3. Identification of ki67-positive neurons in the VTA
a: The representative image of double staining of GAD67 mRNA and ki67. Violet staining indicates GAD67 mRNA. Brown nuclear staining indicates ki67. Black arrows indicate GAD67 mRNA and ki67 double-positive neurons. White arrows indicate GAD67 mRNA-positive, ki67-negative neurons. Image of the rostral region of the VTA. b: Percentage of GAD67 mRNA-positive neurons among ki67-positive neurons. * P < 005. Unpaired t-test. Scale = 10 µm.

GAD67 mRNA staining was performed similarly to the methods described in the main text of the in situ hybridization for GAD67 sections. After in situ hybridization, the sections were washed and incubated with a rabbit anti-ki67 monoclonal antibody (MA5-14520, 1:50, ThermoFisher, IL) in blocking buffer (2% blocking reagent [Roche Applied Science, Upper Bavaria, Germany] in TBS) overnight at 4ºC. The sections were then washed in 0.05 M Tris-HCl buffer (pH 7.5s), and incubated with biotinylated goat anti-mouse IgG (BA-9200, 1:400, VECTOR Laboratories Inc. CA) for 40 min. Again, the sections were washed in 0.05 M Tris-HCl buffer (pH 7.5), then incubated with an avidin-

biotin complex (ABC kit; Vector Laboratories Inc., CA). Next, the sections were washed and immunoreactions were visualized by incubating in diaminobenzidine (DAB) solution without nickel ammonium, and rinsed with Tris-HCl buffer. The sections were mounted on a glass slide and cover-slipped using Entellan new (Merk, Darmstadt, Germany). GAD67 mRNA-positive neurons, ki67-positive neurons, and double-positive neurons were counted under a light microscope.
